# Lung adenocarcinoma with Lambert–Eaton myasthenic syndrome indicated by voltage-gated calcium channel: a case report

**DOI:** 10.1186/1752-1947-6-281

**Published:** 2012-09-05

**Authors:** Hiromasa Arai, Kenji Inui, Kazuki Hashimoto, Kazuki Kan-o, Teppei Nishii, Hitaru Kishida, Koji Okudela, Masahiro Tsuboi, Akinori Nozawa, Takeshi Kaneko, Munetaka Masuda

**Affiliations:** 1 Respiratory Disease Center, Yokohama City University Medical Center, 4-57 Urafune-cho, Minami-ku, Yokohama 232-0024, Japan; 2 Department of Neurology, Yokohama City University Medical Center, 4-57 Urafune-cho, Minami-ku, Yokohama 232-0024, Japan; 3 Department of Pathology, Yokohama City University Medical Center, 4-57 Urafune-cho, Minami-ku, Yokohama 232-0024, Japan; 4 Department of Surgery, Yokohama City University Graduate School of Medicine, 3-9 Fukuura, Kanazawa-ku, Yokohama 236-0004, Japan; 5 Department of Pathology, Yokohama City University Graduate School of Medicine, 3-9 Fukuura, Kanazawa-ku, Yokohama 236-0004, Japan

**Keywords:** Lambert–Eaton myasthenic syndrome, Lung adenocarcinoma, Voltage-gated calcium channel, Immunostaining

## Abstract

**Introduction:**

Lambert–Eaton myasthenic syndrome is a rare disorder and it is known as a paraneoplastic neurological syndrome. Small cell lung cancer often accompanies this syndrome. Lambert–Eaton myasthenic syndrome associated with lung adenocarcinoma is extremely rare; there are only a few reported cases worldwide.

**Case presentation:**

A 75-year-old Japanese man with a past history of chronic rheumatoid arthritis and Sjögren syndrome was diagnosed with Lambert–Eaton myasthenic syndrome by electromyography and serum anti-P/Q-type voltage-gated calcium channel antibody level preceding the diagnosis of lung cancer. A chest computed tomography to screen for malignant lesions revealed an abnormal shadow in the lung. Although a histopathological examination by bronchoscopic study could not reveal the malignancy, lung cancer was mostly suspected after the results of a chest computed tomography and [^18^F]-fluorodeoxyglucose positron emission tomography. An intraoperative diagnosis based on the frozen section obtained by tumor biopsy was adenocarcinoma so the patient underwent a lobectomy of the right lower lobe and lymph node dissection with video-assisted thoracoscopic surgery. The permanent pathological examination was the same as the frozen diagnosis (pT2aN1M0: Stage IIa: TNM staging 7th edition). Immunohistochemistry revealed that most of the cancer cells were positive for P/Q-type voltage-gated calcium channel.

**Conclusions:**

Our case is a rare combination of Lambert–Eaton myasthenic syndrome associated with lung adenocarcinoma, rheumatoid arthritis and Sjögren syndrome, and to the best of our knowledge it is the first report that indicates the presence of voltage-gated calcium channel in lung adenocarcinoma by immunostaining.

## Introduction

Lambert–Eaton myasthenic syndrome (LEMS) is a rare but well-known paraneoplastic disorder characterized by muscle weakness and fatigability predominantly involving the proximal lower limbs. Recently, measurement of serum anti-P/Q-type voltage-gated calcium channel (VGCC) antibody level has been used, in addition to clinical manifestations and nerve conduction studies, to diagnose LEMS. Patients with LEMS have mostly small cell lung cancer (SCLC); other subtypes of lung cancer are extremely rare. We present a rare case of LEMS associated with pulmonary adenocarcinoma, chronic rheumatoid arthritis (RA) and Sjögren syndrome (Sjs) and provide findings of the resected specimen immunostained with P/Q-type VGCC.

## Case presentation

**Figure 1:**
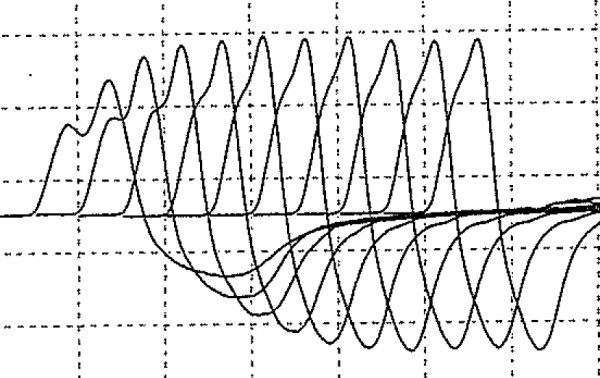
Provocative nerve conduction study of patient’s ulnar nerve showing a trend of increasing at 15Hz of high-frequency stimulation.

The patient was a 75-year-old Japanese man with progressive muscle weakness of his bilateral lower extremities and gait disturbance. He was referred to the Department of Neurology of our hospital for workup. His neurological examination showed muscle weakness and atrophy of his lower extremities, depressed deep tendon reflexes and autonomic dysfunction, that is, dry mouth and orthostatic hypotension. His results for manual muscle strength testing increased from 3 out of 5 to 4– out of 5 for his bilateral lower extremities after a sustained 30-second contraction. His sensation was intact to light touch, and pinprick and Babinski’s sign were down-going bilaterally. A provocative nerve conduction study on the ulnar nerve showed no reduction after repetitive stimulation and tended to increase at 15Hz of high-frequency stimulation (Figure 1). Serum P/Q-type VGCC antibody level was elevated to 31.8pmol/L (cut-off point <20.0pmol/L [1]). Taken together with clinical findings, we made a diagnosis of LEMS. A chest abdominal computed tomography (CT) to screen for malignant lesions revealed an abnormal shadow in the right pulmonary lower lobe. He was then referred to our department (Thoracic Surgery in Respiratory Disease Center) for diagnosis and treatment of lung tumor. 

**Figure 2:**
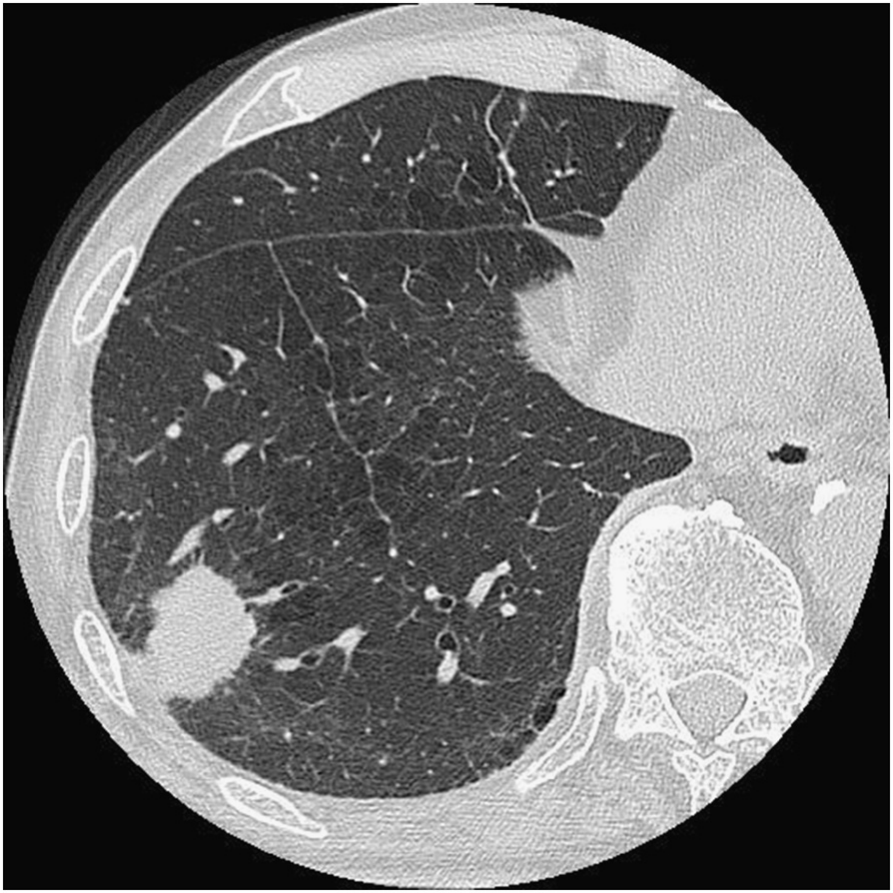
Chest computed tomography scan showing the shadow of a solid mass with spiculation in the patient’s right Segment 9b region, and emphysematous lung.

The lung tumor with spiculation was located in Segment 9b, with a maximum diameter of 30mm (Figure 2). There was no lymphadenopathy or pleural effusion. A CT of the patient’s chest showed a very bilateral emphysematous lung. No abnormality was seen in his abdomen by CT or in the brain by enhanced magnetic resonance imaging (MRI). A [^18^F]-fluorodeoxyglucose positron emission tomography showed increased uptake of fluorodeoxyglucose in the mass region and right hilar region (maximum standardized uptake values were 9.6 and 3.3, respectively). Although a bronchoscopic examination did not reveal malignancy, primary lung cancer was strongly suspected.

The patient had worked as a carpenter before the onset of muscle weakness. However, as his symptoms worsened, he required assistance in daily life (performance status 2). He was admitted to our ward by wheelchair. He had a history of chronic RA medicated by oral prednisolone therapy. He also complained of thirst for many months. A full blood examination and biochemical examination were almost within the normal range. Carcinoembryonic antigen was slightly elevated to 6.0ng/mL, whereas other tumor markers for lung cancer (squamous cell carcinoma-related antigen, sialylated Lewis X-i antigen, cytokeratin fragment, neuron-specific enolase and progastrin-releasing peptide) were within normal ranges. On screening for autoimmune disease and collagen disease, serum CH_50_ was slightly elevated to 46.7U/mL (normal range, 30 to 45), and rheumatoid factor was within the normal range (10.4IU/mL; normal range, 0 to 20). On screening for other collagen diseases, anti-nuclear antibody was positive at the titer of 1:640 and anti-SSA/Ro antibody was elevated to >500U/mL (normal range, 0–100). At this time it was clear that the patient also had Sjs. He had a history of cigarette smoking (40 cigarettes per day for 40 years). The preoperative spirometry result was, %VC: 63.9% and FEV_1.0%_ 76.0%; and the blood gas analysis result was pCO_2_ 38.9mmHg and pO_2_ 83.1mmHg (room air).

An operation was performed for a definite diagnosis and treatment of the tumor. An intraoperative frozen diagnosis obtained by tumor biopsy revealed lung adenocarcinoma, and he underwent right lower lobe lobectomy and lymph node dissection with video-assisted thoracoscopic surgery. The operation took 230 minutes, with a blood loss of 200mL. In the postoperative course, because of prolonged air leakage from the remaining right pulmonary lobes, tube drainage lasted for 9 days. Muscle weakness gradually improved on approximately postoperative day 5, and he could walk without assistance. Serum P/Q-type VGCC antibody level was still elevated to 30.6pmol/L on postoperative day 7. He underwent daily rehabilitation for discharge. Approximately 2 months after the operation, he had aspiration pneumonia with respiratory failure. Intubation and respiratory management were performed in the intensive care unit. He remains ventilator dependent at 4 months postoperation. Postoperative provocative nerve conduction studies could not be performed.

**Figure 3:**
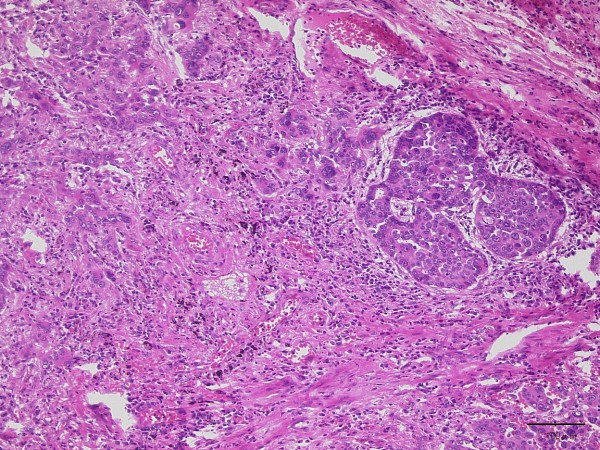
**Microscopic findings of lung adenocarcinoma.** The lesion shows proliferation of infiltrating atypical epithelial cells with cribriform or papillary structures, consistent with adenocarcinoma (hematoxylin-eosin stain).

**Figure 4:**
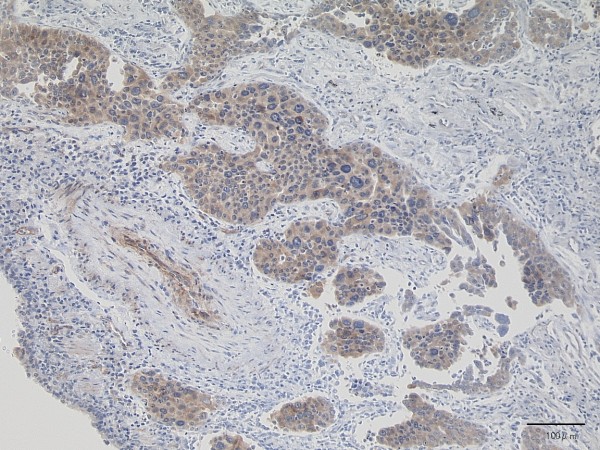
Most of the cancer cells are positive for P/Q-type VGCC (immunohistochemistry).

His tumor was composed of a proliferation of infiltrating atypical cells with cribriform or papillary structures. The tumor measured 30mm in size and the diagnosis was moderately-differentiated (G2) mixed-type adenocarcinoma (papillary and acinar adenocarcinoma) (Figure 3). Lymph nodes metastases in the lung parenchyma were noted. Pathological T2aN1M0 (Stage IIa: TNM staging 7th edition) was diagnosed. Immunohistochemistry revealed that most of the cancer cells were positive for P/Q-type VGCC (Sigma-Aldrich®; St. Louis, MO, USA) (Figure 4).

## Discussion

LEMS is one of the representative paraneoplastic syndromes and an autoimmune disease of the neuromuscular junction. Because of its scarcity, the exact incidence of LEMS is unknown. The prevalence of as few as 400 cases in the USA in 1994 [2] and of 2 to 3 cases per million inhabitants in the Netherlands in 2004 [3] has been reported.

LEMS is known to be associated with malignant neoplasm, especially SCLC. Approximately 50 to 75% of patients with LEMS have SCLC [2-7].

LEMS is also thought to be a complication of organ-specific autoimmune diseases, such as thyroid disease, vitiligo, pernicious anemia, coeliac disease, and juvenile-onset diabetes mellitus. However, systemic lupus erythematosus, Sjs and RA associated with LEMS is rare; only a few cases are reported in the English literature [4,8].

Clinical manifestation of LEMS is usually characterized by proximal muscle weakness affecting the legs more than the arms, fatigability and dysautonomia. Malignant neoplasm usually occurs within the first 2 years after the onset of LEMS and in almost all patients after 4 years [5]. Therefore, once LEMS is diagnosed or strongly suspected, screening for malignant neoplasm, especially SCLC (for example, using chest CT or tumor markers: neuron-specific enolase and progastrin-releasing peptide), is recommended. Moreover, careful observation and screening should be required for at least 2 years [4-6]. The treatment of SCLC controls its mortality and often results in normalization of both electrodiagnostic findings and clinical symptoms of LEMS [5,6].

The diagnosis of LEMS is established by clinical manifestation and by characteristic findings on nerve conduction studies. The nerve conduction study findings indicate reduced amplitude of compound muscle action potential that increases by over 100% after maximum voluntary activation or 20 to 50Hz of nerve stimulation, and incremental response (waxing) of compound muscle action potential on the rapid rate of repetitive stimulation [5,6].

In the 1990s, Motomura and co-workers established a new immunoassay of antibodies to VGCC for the diagnosis of LEMS [1]. VGCCs are situated on the presynaptic nerve terminal and have a central role in the control of neurotransmitter release [1,9]. Antibodies to VGCC inhibit transmitter (acetylcholine) release at presynaptic nerve terminals. It has been assumed that SCLC expresses functional calcium channels and that autoantibodies to these channels may cross-react with similar VGCCs [1,5,6,9]. Motomura [1] reported 85% of patients with LEMS to be positive for VGCC and suggests that the titer of anti-P/Q-type VGCC antibody in particular occupies an important position in the diagnosis of LEMS; the remaining 15% of patients were seronegative for VGCC [9]. In this subgroup, SCLC is less common and the exact explanation of seronegative LEMS is still unknown. Thus, a negative antibody titer of VGCC is not necessarily an indicator of the absence of LEMS [9].

LEMS is rarely associated with other subtypes of lung cancer like adenocarcinoma. A PubMed literature search (keywords, ‘Lambert-Eaton Myasthenic Syndrome’ and ‘lung adenocarcinoma’) found only two publications in the English literature [7,10]. In Japan, although five cases have been reported to date, there are only two reports of LEMS associated with lung adenocarcinoma in Japanese research with an English abstract [11,12]. The clinical features of these five cases, including our case report, are summarized in Table 1. The patients (all men) ranged from 32 to 75 years of age (mean age, 57 ± 16.0 years). Adenocarcinomas arose in the right lung in all cases. Coexistent collagen diseases were recognized in two cases, including our case (dermatomyositis, RA and Sjs). Three out of these five cases could undergo lung resection. Almost all LEMS cases are related to SCLC, making them unsuitable for resection. For cases not associated with SCLC, resection is a possibility. Therefore, once LEMS is strongly suspected, screening for malignancy and early detection of lung carcinoma and its histological type is very important. Of note, the histological differentiation of all demonstrated cases was poor to moderate. 

**Table 1:** Characteristics of patients with Lambert–Eaton myasthenic syndrome associated with lung adenocarcinoma

**Case no.**	**Year**	**First author**	**Age**	**Sex**	**Site**	**Histological grade**	**Coexistent collagen disease**	**Treatment**	**Outcome**
1	1987	Ramos-Yeo [7]	56	M	RLL	Poor	None	Steroid, plasmapheresis	Death at 2 years
									(due to infection)
2^a^	1989	Sumitomo [11]	58	M	RML	Poor	None	Lobectomy,	Surviving at 2 years
								adjuvant chemotherapy	
3^a^	1996	Okudera [12]	32	M	RUL	Poor	None	Chemotherapy	Death at 5 months
									(due to DIC)
4	2008	Milanez [10]	66	M	RUL	Moderate	DM	Lobectomy	Death at 16 days
									(due to sepsis)
5	2012	This case report	75	M	RLL	Moderate	RA, Sjs	Lobectomy	Surviving at 4 months

a: English abstract only; *DIC* disseminated intravascular coagulation, *DM* dermatomyositis, *RA*, rheumatoid arthritis, *RLL* right lower lobe, *RML* right middle lobe, *RUL* right upper lobe, *Sjs* Sjögren syndrome.

We examined P/Q-type VGCC expression immunohistochemically using resected lung tumor sections. Most of the neoplastic cells were stained positive, and the area consisting of poorly-differentiated neoplastic cells tended to show stronger signals. As far as we know, no report has demonstrated immunohistochemical expression of VGCC in neoplastic cells.

## Conclusions

Taking together the results of immunostaining and the fact that our patient also had collagen diseases (RA and Sjs), autoimmune function may be related to the onset of LEMS and, in the case of lung adenocarcinoma, lower-differentiated cases appear to develop P/Q-type VGCC. However, the actual mechanism by which anti-P/Q-type VGCC antibody causes LEMS is unclear because the experimental model of this rare disease has not been established [6]*.* Further molecular biological studies are required. Clinically, physicians must manage patients with muscle weakness while considering LEMS as a differential diagnosis. Recognition and awareness of this rare syndrome are essential for a better prognosis and quality of life for these patients.

## Consent

Written informed consent was obtained from the patient of this case report and any accompanying images. A copy of the written consent is available for review by the Editor-in-Chief of this journal.

## Competing interest

The authors declare that they have no competing interests.

## Authors’ contributions

HA was responsible for the conception and writing of the manuscript. KH, KK and TN participated in the collection of data and patient care. HK, KO and AN provided collection of data, patient care and editing of the manuscript. KI, MT, TK and MM participated in final revision of the manuscript and guidance. All authors read and approved the final manuscript.
